# Military Surgery

**Published:** 1916-11

**Authors:** 


### Military Surgery.

**Duodenal Ulceration,** general peritonitis from perforation, death. Merklen and Jourdan, independently report a case each, occurring in soldiers and seen in the hospital only in the terminal stage. It is implied that, in civil practice, better opportunities would have been presented for prompt diagnosis and therapeutic measures. A classic epigram by Grisolle is quoted to the effect that peritonitics die talking, meaning that the end comes suddenly without unconsciousness. In the discussion (Medical Reunion of the 4th Army), Potherat called attention to the fact that juxta-pyloric ulcers are rare in military experience and that this is not entirely due to the exclusion of women (nor to the superior physical condition of soldiers and their age) as they are not even common in the zone of military occupation.

